# Alterations in Antibiotic Susceptibility of *Staphylococcus aureus* and *Klebsiella pneumoniae* in Dual Species Biofilms

**DOI:** 10.3390/ijms24108475

**Published:** 2023-05-09

**Authors:** Anna V. Mironova, Agniya V. Karimova, Mikhail I. Bogachev, Airat R. Kayumov, Elena Y. Trizna

**Affiliations:** 1 Institute of Fundamental Medicine and Biology, Kazan Federal University, 420008 Kazan, Russia; 2 Biomedical Engineering Research Centre, St. Petersburg Electrotechnical University, 197022 St. Petersburg, Russia

**Keywords:** dual-species biofilms, sensitivity to antimicrobials, extracellular matrix, biochemical composition, gene expression

## Abstract

In the last decades, it has been shown that biofilm-associated infections in most cases are caused by rather two or even more pathogens than by single microorganisms. Due to intermicrobial interactions in mixed communities, bacteria change their gene expression profile, in turn leading to alterations in the biofilm structure and properties, as well as susceptibility to antimicrobials. Here, we report the alterations of antimicrobials efficiency in mixed biofilms of *Staphylococcus aureus*–*Klebsiella pneumoniae* in comparison with mono-species biofilms of each counterpart and discuss possible mechanisms of these alterations. In cell clumps detached from dual-species biofilms, *S. aureus* became insensitive to vancomycin, ampicillin, and ceftazidime compared to solely *S. aureus* cell clumps. In turn, the increased efficiency of amikacin and ciprofloxacin against both bacteria could be observed, compared to mono-species biofilms of each counterpart. Scanning electron microscopy and confocal microscopy indicate the porous structure of the dual-species biofilm, and differential fluorescent staining revealed an increased number of polysaccharides in the matrix, in turn leading to more loose structure and thus apparently providing increased permeability of the dual-species biofilm to antimicrobials. The qRT-PCR showed that *ica* operon in *S. aureus* became repressed in mixed communities, and polysaccharides are produced mainly by *K. pneumoniae*. While the molecular trigger of these changes remains undiscovered, detailed knowledge of the alterations in antibiotic susceptibility to given drugs opens doors for treatment correction options for *S. aureus*–*K. pneumoniae* biofilm-associated infections.

## 1. Introduction

Biofilm formation by pathogenic bacteria represents an acute challenge in infectious medicine because of the extreme tolerance of biofilm-embedded bacteria to antimicrobials [1,2,3,4]. A significant increase in resistance is primarily provided by the extracellular polymer matrix, the components of which are produced by microorganisms themselves [2,5,6]. It consists of polysaccharides, proteins, and DNA in various ratios, forming a diffusional barrier preventing the penetration of various adverse environmental factors such as antimicrobials, metal ions, and the immune system of the host [7,8,9,10].

Mixed biofilms formed under natural conditions typically consist of several types of various microorganisms [6]. Interspecies microbial interactions in such communities may be either synergistic or antagonistic, and this may influence the course, treatment, and outcome of a disease associated with a multi-species infection [11,12]. Moreover, in multi-species communities, changes in the metabolic profile of the cell lead to alterations in both structure and properties of the biofilm [13,14]. In particular, either activation or repression of virulence factors and associated changes in the sensitivity to antimicrobial therapy may occur [15]. Quorum sensing also plays a major role in bacterial pathogenesis, self-defense via biofilm formation, and inter-species communication [16,17]. Many investigations report that bacterial susceptibility to antimicrobials drastically changes in mixed communities. Thus, in *Staphylococcus aureus*–*Pseudomonas aeruginosa* the sensitivity of each counterpart to aminoglycosides increases [18,19], while *S. aureus* becomes insusceptible to vancomycin because of the action of various metabolites secreted by *P. aeruginosa* [20]. 

*Klebsiella pneumoniae* is an opportunistic pathogen that commonly causes nosocomial infections in the urinary tract, respiratory tract, lungs, and acute wounds [21]. Genomic analysis of *K. pneumoniae* isolates revealed a wide range of genetic variability, including multidrug resistance and hypervirulence [22]. The ability of *K. pneumoniae* to form mixed-species biofilm raises additional challenges in the treatment of infections, especially chronic wounds and ventilator-associated infections. While third-generation cephalosporins, fluoroquinolones, carbapenems, and aminoglycosides are widely used to treat *K. pneumoniae* infections [23,24,25], the bacterial susceptibility to treatment significantly changes for biofilms. Tanner et al. showed that a plasmid carrying the carbapenemase resistance gene (*bla*_NDM-1_) can be transferred from *Escherichia coli* to *P. aeruginosa* and *Acinetobacter baumannii,* by conjugation in di-microbial biofilms [26]. Thuy et al. screened *S. aureus* and *K. pneumoniae* for virulence genes and showed a preponderance of multidrug-resistant *S. aureus* clones and a few hypervirulent *K. pneumoniae* clones among patients [27]. Furthermore, in the multi-species biofilm consisting of *P. aeruginosa*, *S. aureus*, *K. pneumoniae,* and *A. baumannii* resistance to ciprofloxacin increased mainly due to the tolerance of *K. pneumoniae*, while monocultures were sensitive to this antibiotic [28]. Thus, while antibiotics of the β-lactam, glycopeptide, tetracycline, macrolide, and lincosamide classes are conventional for *S. aureus* treatment, their efficiency in mixed communities changes [29,30,31]. For example, the increased resistance at the mixed community level of *P. aeruginosa*, *P. protegens,* and *K. pneumoniae* was observed with tobramycin and SDS treatment [32]. At the same time, resistance was not the result of the selection of non-susceptible species over other more susceptible ones but rather was a cross-protection offered by resistant species to all members of the community [33].

However, to date, studies of interspecies interactions in *S. aureus*–*K. pneumoniae* biofilms are only scarcely represented, and the mechanisms of their mutual inference remain almost unelaborated. Understanding these interactions will help to find ways to prevent and treat chronic *K. pneumoniae* infections, and thus potentially facilitate the development of novel antibacterial agents against biofilm-associated infections. 

Here, we show that *S. aureus*–*K. pneumoniae* dual-species biofilms are characterized by the nearly two-fold increased number of polysaccharides produced mainly by *K. pneumoniae* in the matrix, that in turn leads to more loose structure of the biofilm compared to mono-species ones. In addition, while in cell clumps detached from dual-species biofilms *S. aureus* became insensitive to ampicillin, vancomycin, and ceftazidime, dual-species biofilms are more permeable for ciprofloxacin, thus providing increased susceptibility of biofilm-embedded bacteria to this antimicrobial. 

## 2. Results

### 2.1. S. aureus and K. pneumoniae Susceptibility to Antibiotics in Mixed Cultures

**Figure 1:**
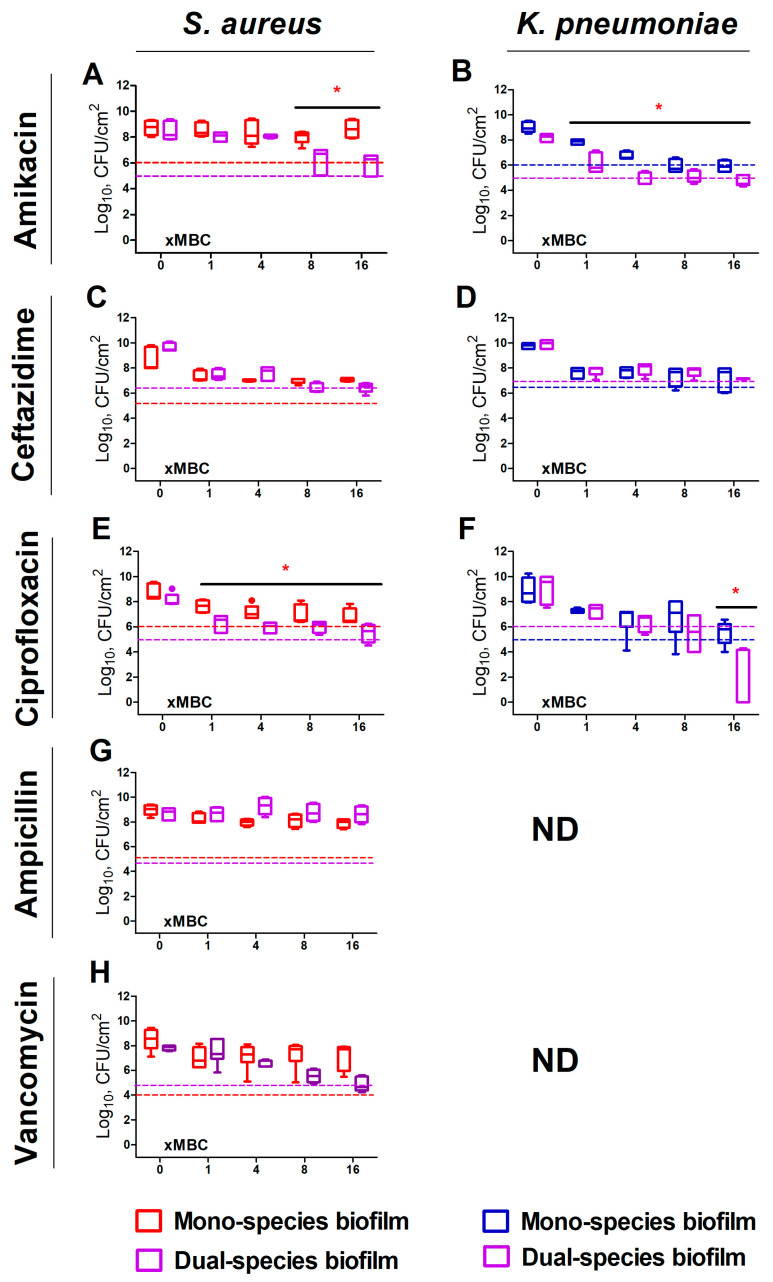
The effect of various antimicrobials on the viability of biofilm-embedded cell *S. aureus* (**A**,**C**,**E**,**G**,**H**) and *K. pneumoniae* (**B**,**D**,**F**) in mono-(red and blue boxes, respectively), and dual-species (violet boxes) cultures. Biofilms were grown for 48 h under static conditions, washed and antimicrobials were added. After 24 h of incubation, the biofilms were washed, adhered cells were scratched, suspended, and CFUs were counted by plating of 10-fold dilutions. Median values with interquartile ranges from five independent measurements are shown. Dotted lines show the level corresponding to the reduction in the CFUs count by 3 orders of magnitude (death of 99.9% of cells). ND indicated non-determined cases. Asterisks indicate statistically significant in CFUs count in treated mono- and dual-species biofilms according to the non-parametric one-way analysis of variance (Kruskal–Wallis) test, * *p* < 0.05.

Figure 1 compares the effects of various antimicrobials on the viability of biofilm-embedded *S. aureus* and *K. pneumoniae* cells in mono- and dual-species cultures. The effect of ampicillin and vancomycin on *K. pneumoniae* was not evaluated because of their low efficiency against this bacterium (see Table 1 for MIC and MBC values). Twenty-four-hour treatment with all antimicrobials was inefficient against biofilm-embedded *S. aureus* in mono-culture (Figure 1A,E (red symbols)) since the criteria of 1000-fold reduction of CFUs has not been fulfilled. *K. pneumoniae* appeared more susceptible, and the CFUs count in biofilm was reduced by three orders of magnitude at 8×MBC of amikacin (Figure 1B (blue symbols)), 16×MBC of ceftazidime and ciprofloxacin (Figure 1D,F (blue symbols)). 

By contrast, both bacteria became more susceptible to treatment in dual-species biofilms. Thus, the three-log reduction of *S. aureus* in mixed biofilm could be achieved at 8×MBC of amikacin and ceftazidime (Figure 1A,C (violet symbols)), as well as at 16×MBC of ciprofloxacin and vancomycin (Figure 1E,H (violet symbols)). Furthermore, the CFUs count of *K. pneumoniae* decreased by three orders of magnitude at 4×MBC of amikacin (Figure 1B (violet symbols)). The bactericidal effect of ciprofloxacin has been observed also at 4×MBC, and almost complete death of bacterium could be achieved at 16×MBC (Figure 1F (violet symbols)). 

**Figure 2:**
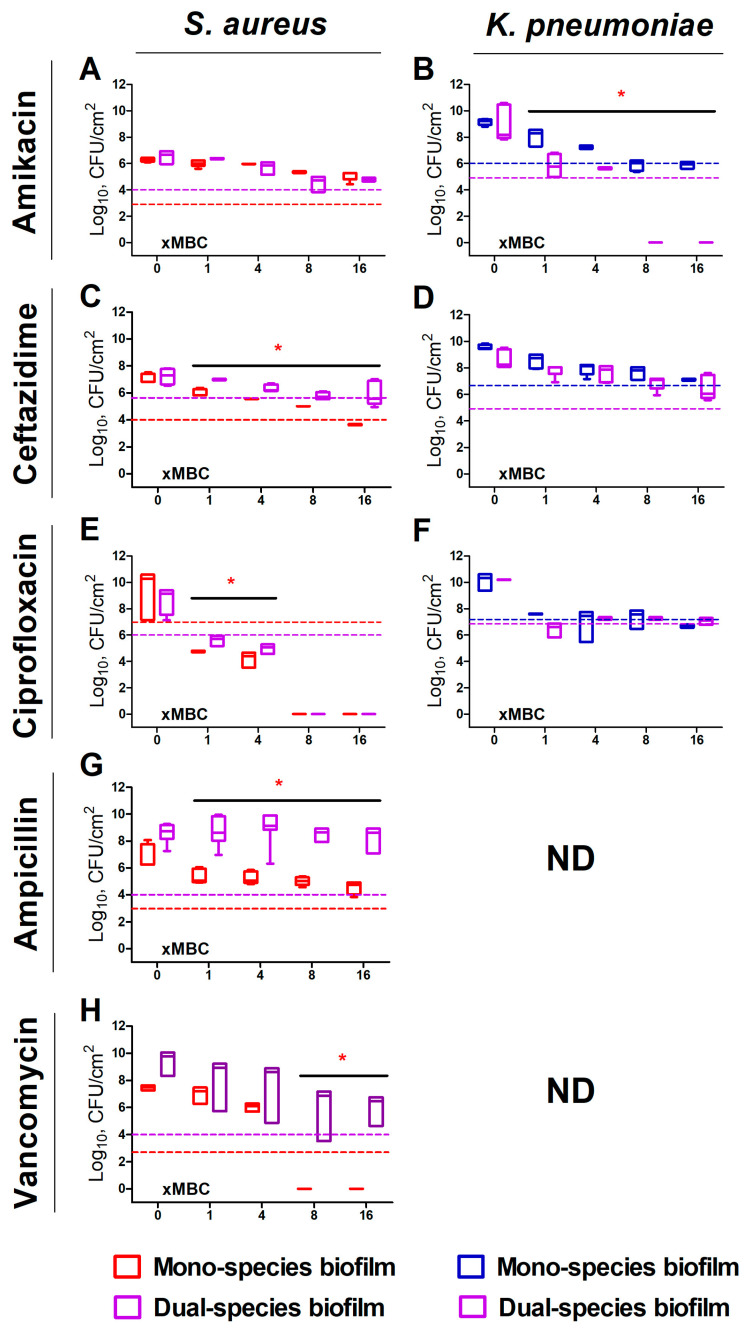
The effect of various antimicrobials on the viability of biofilm-detached cell clumps of *S. aureus* (**A**,**C**,**E**,**G**,**H**), and *K. pneumoniae* (**B**,**D**,**F**) in mono- (red and blue boxes, respectively), and dual-species (violet boxes) cultures. Biofilms were grown for 48 h under static conditions, washed and antimicrobials were added. After 24 h of incubation, the biofilms were washed, adhered cells were scratched, suspended, and CFUs were counted by plating of 10-fold dilutions. Median values with interquartile ranges from five independent measurements are shown. Dotted lines show the level corresponding to the reduction in the CFUs count by 3 orders of magnitude (death of 99.9% of cells). ND denotes undetermined cases. Asterisks indicate statistically significant in CFUs count in treated mono- and dual-species biofilms according to the non-parametric one-way analysis of variance (Kruskal–Wallis) test, * *p* < 0.05.

**Figure 3:**
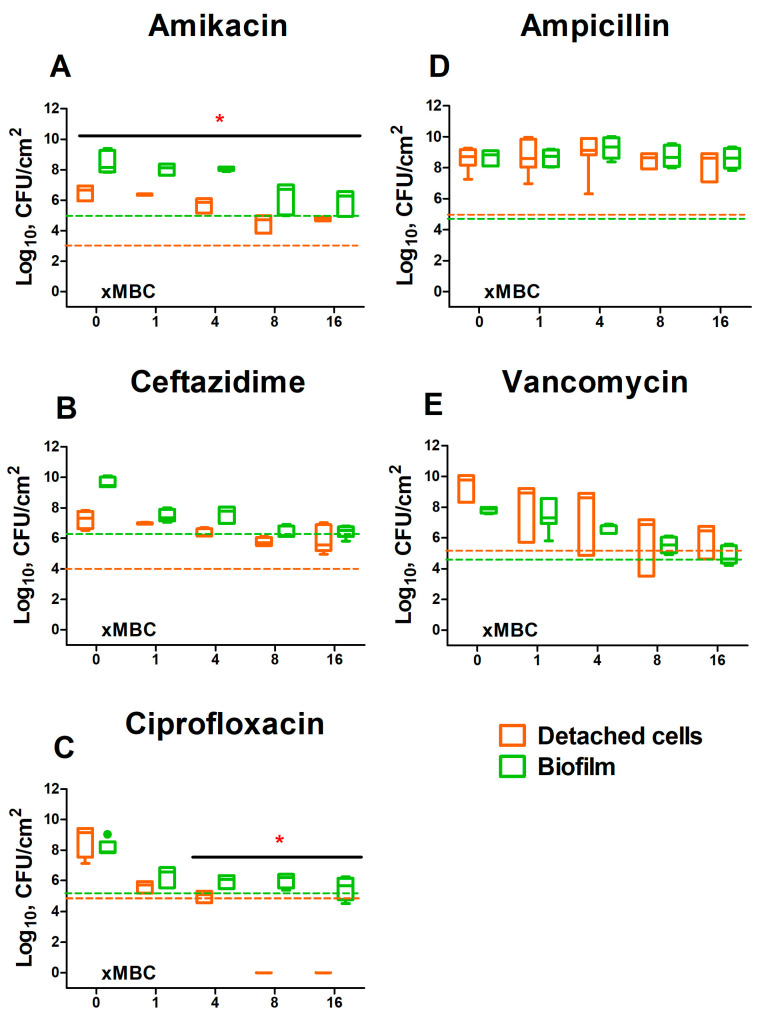
The comparison of susceptibility to various antimicrobials (**A**–**E**) of *S. aureus* in biofilm and in detached cell clumps in *S. aureus–K. pneumoniae* mixed culture. Biofilms were grown 48 h under static conditions, washed and antimicrobials were added. After 24 h of incubation, CFUs were counted in both the biofilm and culture liquid by plating of 10-fold dilutions. Median values with interquartile ranges from five independent measurements are shown. Dotted lines show the level corresponding to the reduction in the CFUs count by 3 orders of magnitude (death of 99.9% of cells). Asterisks indicate statistically significant in CFUs count in treated mono- and dual-species biofilms according to the non-parametric one-way analysis of variance (Kruskal–Wallis) test, * *p* < 0.05.

In the detached cell clumps (Figure 2), *K. pneumoniae* susceptibility to both amikacin and ciprofloxacin increased significantly, indicated by three-log reduction of CFUs has been observed at 1×MBCs (Figure 2B,F (violet symbols)), while ceftazidime did not demonstrate similar effect (Figure 1D (violet symbols)). Surprisingly, *S. aureus* in cell clumps detached from the dual-species biofilm, exhibited a decreased susceptibility to all antimicrobials excepting amikacin (Figure 2A (violet symbols)). Furthermore, the decrease rate of viable *S. aureus* cells in both biofilm and cell clumps were the same, indicating that the presence of *K. pneumoniae* significantly affects the susceptibility of *S. aureus* to antimicrobials (Figure 3).

### 2.2. Evaluation of the Permeability of S. aureus and K. pneumoniae Mono- and Dual-Species Biofilms for Antimicrobials

Observed changes in *S. aureus* and *K. pneumoniae* susceptibility to antibiotics in both biofilms and detached cell clumps of mixed communities allowed suggesting that this effect is apparently governed by the alterations either in the composition or in the structure of the biofilm matrix. In order to check this assumption, a comparative assessment of the permeability of mono- and dual-species biofilms for antibiotics was carried out.

**Figure 4:**
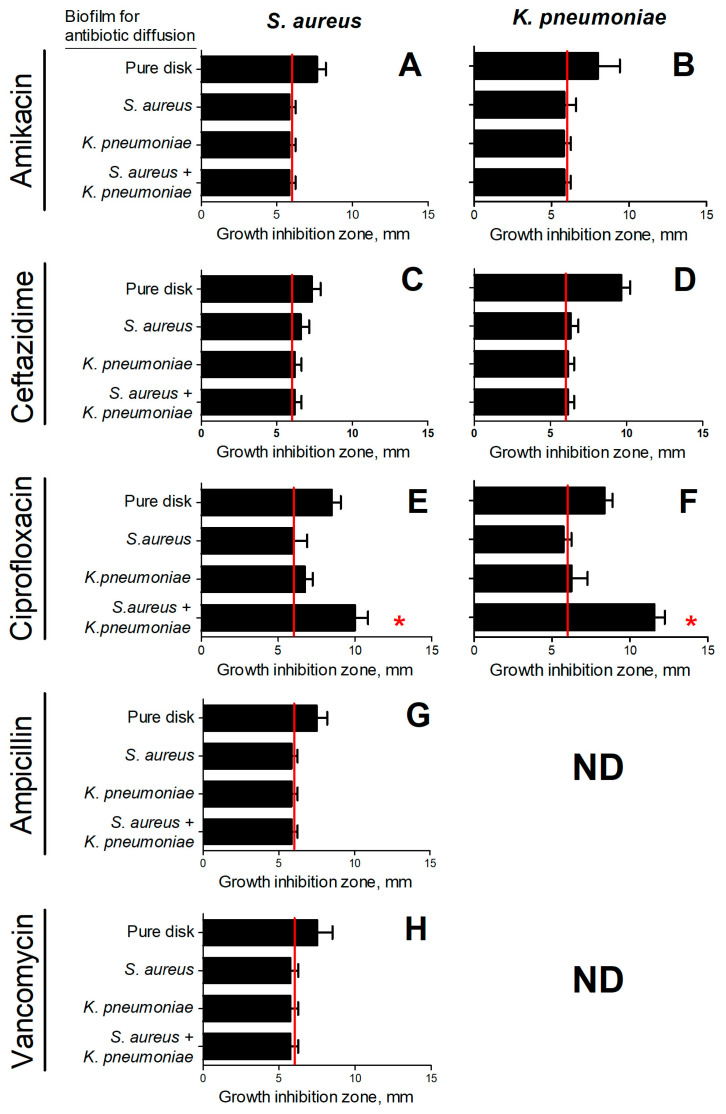
The permeability of *S. aureus* and *K. pneumoniae* dual-species biofilm compared to monospecies biofilms of both counterparts. The 48 h old biofilms were established on nitrocellulose discs and then disks were placed on agar containing an antibiotic at a concentration corresponding to 1×MBC in relation to the studied bacteria. The biofilm was covered with another nitrocellulose disc and Whatmann 3M disk was placed to allow diffusing of an antibiotic through biofilm and accumulate in the paper disk. After 24 h, paper disks were transferred to plates with bacterial lawns, and growth inhibition zones of *S. aureus* (**A**,**C**,**E**,**G**,**H**) and *K. pneumoniae* (**B**,**D**,**F**) were measured after the next 24 h growth. As a control, all manipulations were performed with the disk without an established biofilm. ND denotes undetermined cases. Asterisks indicate statistically significant differences according to the non-parametric one-way analysis of variance (Kruskal–Wallis) test, * *p* < 0.05.

As can be seen from Figure 4, in the case of ciprofloxacin, the growth inhibition zone of both bacteria around disks taken from mixed biofilms was significantly larger compared to those from mono-species biofilms, and similar to control disks. This indicates that mixed biofilm of *S. aureus*–*K. pneumoniae* became more permeable for fluorchinolone than monocultural biofilm. Ceftazidime could slightly diffuse through *S. aureus* biofilm, while both *K. pneumoniae* and *S. aureus*–*K. pneumoniae* biofilms remain non-permeable for this antibiotic (Figure 4C,D).

Almost no growth inhibition zone of either of the studied bacteria could be observed in the case of amikacin, even in control (Figure 4A,B). Apparently, this antimicrobial diffuses too slowly in this model. By contrast, as could be seen from Figure 4G,H *S. aureus* biofilms are non-permeable for ampicillin and vancomycin.

### 2.3. The Biochemical Composition of Extracellular Matrix in S. aureus and K. pneumoniae Mono- and Dual-Species Biofilms 

**Figure 5:**
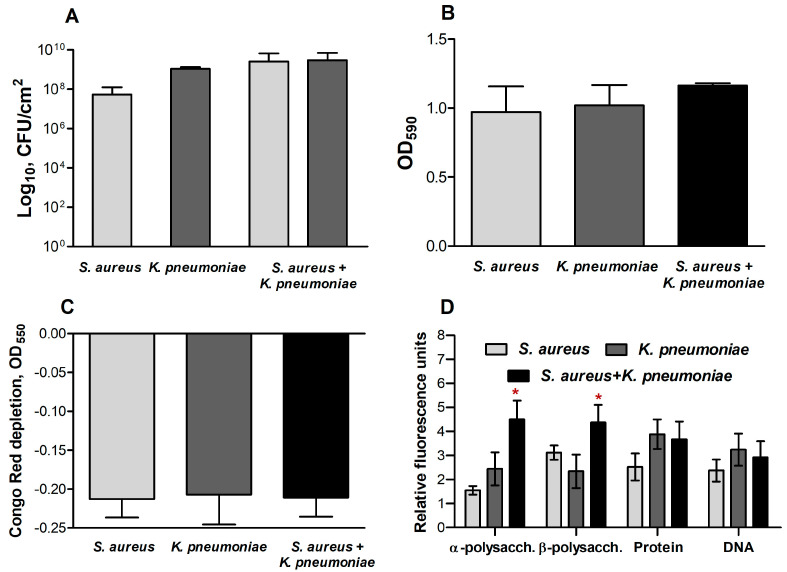
Comparative analysis of CFUs number (**A**), total biomass (**B**), extracellular matrix mass (**C**), and matrix components (**D**) in *S. aureus* and *K. pneumoniae* mono- and dual-species biofilms. Bacterial biofilms were grown in 24-well adhesive plates for 48 h at 37 °C under static conditions. CFUs were counted by plating of 10-fold dilutions of scratched biofilm (**A**); the masses of the biofilm (**B**) and of the extracellular matrix (**C**) were quantified by crystal violet and Congo red staining, respectively. DNA, proteins, α- and β-polysaccharides of the matrix (**D**) were differentially stained with SYBR Green, Sypro Orange, ConA-TMR, CFW, respectively. Asterisks indicate statistically significant differences according to non-parametric one-way analysis of variance (Kruskal–Wallis) test, * *p* < 0.05.

In order to check for possible alterations in the extracellular matrix content and composition in mono- and dual-species biofilms, the total biomass and mass of the extracellular matrix were assessed in *S. aureus* and *K. pneumoniae* mono- and dual-species biofilms. For that, 48 h mono- and dual-species biofilms were stained with either crystal violet (stains the entire biomass of the biofilm) or Congo red (stains mainly the extracellular matrix). In mixed *S. aureus*–*K. pneumoniae* biofilm, the viable cells count of either bacteria was the same (Figure 5A), without any significant differences in total and matrix biomasses between mono- and dual-species biofilms observed (Figure 5B,C). 

Further, the biochemical composition of both mono- and dual-species biofilm matrixes of *S. aureus* and *K. pneumoniae* have been assessed with differential fluorescent staining with SYBR Green, Sypro Orange, ConA-TMR, and CFW fluorescent dyes, which bind to DNA, proteins, α-and β-polysaccharides, respectively.

In the dual-species community, a significant increase in the number of polysaccharides compared to monomicrobial biofilms of respective counterparts has been observed (Figure 5D). At the same time, the amount of extracellular DNA and proteins in the mixed biofilm did not differ significantly from monocultures, indicating no apparent alterations in the accumulation of the above components.

### 2.4. Microscopy

**Figure 6:**
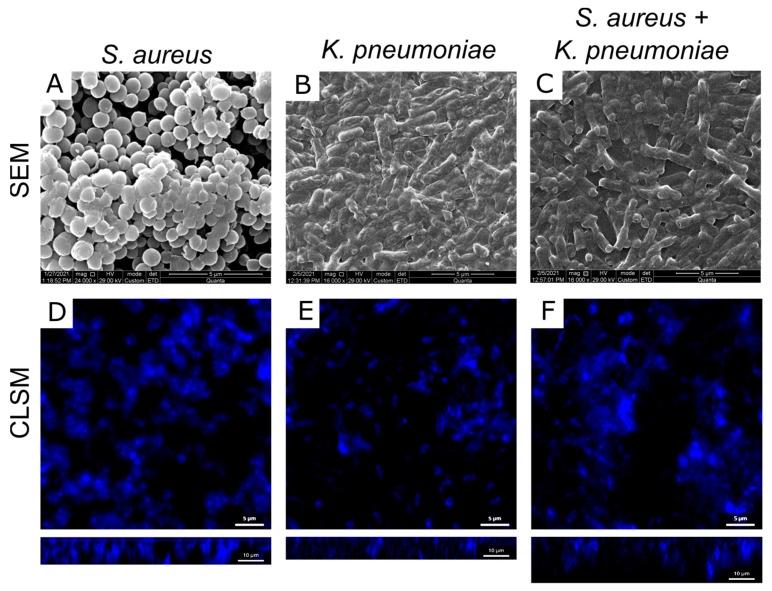
Comparative analysis of the ultrastructure of *S. aureus* and *K. pneumoniae* mono- and dual-species biofilms. The 48 h old biofilms were analyzed with scanning electron microscopy (SEM) with magnification of 16,000× (**A**–**C**) or stained with DAPI and analyzed with confocal laser scanning microscopy (CLSM) on an Olympus IX83 inverted microscope with magnification of 100× (**D**–**F**) to visualize the biofilm thickness.

In the next step, the structure, cell distribution, and biochemical composition of the extracellular matrix of both mono- and dual-species biofilms have been analyzed with SEM and CLSM. As could be seen from the SEM microphotographs, the biofilm structure demonstrated alterations in the mixed community in comparison with the biofilms of their counterparts. Thus, the biofilm structure becomes looser with multiple pores and holes compared to *K. pneumoniae* biofilm, while more extracellular matrix could be observed compared to *S. aureus* biofilm (Figure 6A–C). Further, to stain individual cells and thus evaluate their distribution density in the biofilm matrix, biofilms were stained with DAPI and analyzed with CLSM. The reconstruction of Z-stacks of mono- and mixed *S. aureus*-*K. pneumoniae* biofilm from data confirmed its loose structure compared to monoculture biofilms, although at the same time, a significant increase in the biofilm thickness has been observed (Figure 6D–F).

**Figure 7:**
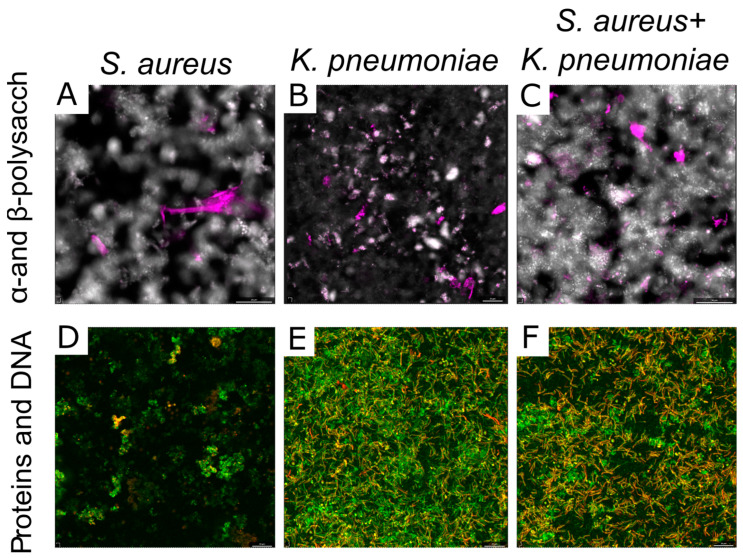
Distribution of the main components of mono- (**A**,**B**,**D**,**E**), and dual-species (**C**,**F**) biofilm matrix formed by *S. aureus* and *K. pneumoniae*. DNA (green), proteins (orange), α- and β-polysaccharides (pink and white, respectively), of the matrix of 48 h old biofilms were differentially stained with SYBR Green, Sypro Orange (**D**–**F**), ConA-TMR, CFW (**A**–**C**), respectively. The microscopy was performed on an Olympus IX83 inverted microscope with magnification of 20×.

As well, differential fluorescent staining with ConA-TMR and CFW confirmed a significant increase in the number of polysaccharides of extracellular matrix in the dual-species community compared to monospecies biofilms (Figure 7A–C). Of note, in the *S. aureus* biofilm mainly α-polysaccharides are detected being localized as separate clusters, while in the *K. pneumoniae* biofilm β-polysaccharides dominate with more homogeneous distribution. In *S. aureus*–*K. pneumoniae* biofilm both α-polysaccharides and β-polysaccharides could be observed and appear rather evenly distributed which could serve as a scaffold for bacterial biofilms with porous structure.

Neither protein nor eDNA content changes in dual-species biofilm could be detected with CLSM (Figure 6D–F). The microphotographs show that proteins and extracellular DNA in the *S. aureus* biofilm are distributed unevenly and are visualized in clusters of different sizes, which is typical for the morphology of *S. aureus* biofilms with a mushroom shape. At the same time, in the *K. pneumoniae* biofilm, proteins and DNA were distributed evenly throughout the sample volume, while in the mixed community, some porosity was observed in the matrix structure with voids between the components significantly smaller compared to the *S. aureus* biofilm (Figure 7).

### 2.5. The Expression of Genes Involved in the Synthesis of the Extracellular Matrix in Mono- and Dual-Species Biofilms of S. aureus–K. pneumoniae

Next, we corroborate the hypothesis that the observed increase in the total number of polysaccharides in *S. aureus–K. pneumoniae* dual-species biofilms may originate from the increase in the synthesis of polysaccharides in a mixed culture either by one of the counterparts or by both of them. To test this assumption, the expression of the *icaA* gene associated with the synthesis of polysaccharide intercellular adhesin (PIA), the dominant component of the *S. aureus* biofilm matrix, as well as the expression of the *pgaA* gene responsible for the *K. pneumoniae* capsule exopolysaccharides synthesis, have been comparatively assessed by reverse transcription polymerase chain reaction in mono- and dual-species biofilms. Since the microcolonies formation as biofilm precursors occur on 10–15 h of growth [34,35] and the *ica* gene exhibited maximum expression on the 8th hour of growth [36], genes expression has been evaluated on the 12th hour of growth (biofilm formation start).

**Figure 8:**
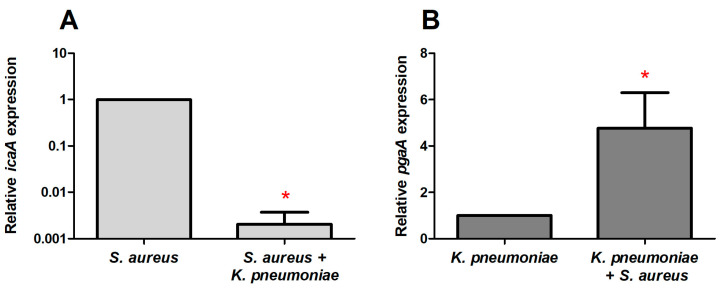
Relative expression of *icaA* (**A**) and *pgaA* (**B**) genes in mono- and dual-species biofilms of *S. aureus* and *K. pneumoniae*. Bacterial biofilms were grown at 37 °C on BM medium in polystyrene adhesive dishes (Eppendorf). The 12 h old biofilms were harvested and total RNA was isolated. The qRT-RCR was performed in one-step approach with SYBR Blue with fluorescent detection of the product using FAM filterset. The 16s rRNA gene was used as reference for *S. aureus* and *proC* gene for *K. pneumoniae*. Relative expression of genes in mono-species biofilms was considered as 1×. Asterisks indicate statistically significant differences according to the non-parametric one-way analysis of variance (Kruskal–Wallis) test, * *p* < 0.05.

As could be seen from Figure 8, the expression level of the *icaA* was reduced 1000-fold in the *S. aureus*–*K. pneumoniae* dual-species biofilm compared to the monoculture of *S. aureus* (Figure 8A). By contrast, the expression of the *pgaA* was four-fold higher in dual-species biofilm, indicating that the synthesis of polysaccharides is apparently provided by *K. pneumoniae* in mixed culture (Figure 8B). 

## 3. Discussion

Being embedded into the biofilm, bacteria become extremely resistant to antibiotics, biocides, and the immune system of the host organism [3,32,37]. The decrease in the sensitivity of bacteria in biofilms is governed by many factors, such as the low permeability of the extracellular matrix, the adaptation of cells to stress, and the transition to a dormant state, due to the limited amount of nutrients and oxygen in the lower layers of the biofilm [22,38]. In the last decade, many investigators have focused on drastic changes in bacterial susceptibility to antimicrobials in mixed communities governed by both interbacterial interactions and biofilm structure alterations. 

Here, we report the profile of antimicrobials efficiency alterations against *S. aureus* and *K. pneumoniae* in mixed biofilms in comparison with monospecies ones of each counterpart and discuss possible mechanisms of these effects. As expected, both bacteria in monoculture biofilms exhibited low sensitivity to various classes of antimicrobials (Figure 1). Nevertheless, in the mixed biofilm, an increase in bacterial sensitivity to amikacin and ciprofloxacin has been observed (Figure 1A,B,E,F), as it has been previously shown for these antimicrobials on *P. aeruginosa–S. aureus* consortium [18]. While it has been demonstrated that various metabolites such as HQNO (2-heptyl-4-hydroxyquinoline-N-oxide, an inhibitor of the electron transport chain), rhamnolipids and siderophores synthesized by *P. aeruginosa* affect the *S. aureus* susceptibility to antimicrobials [20,39], no relevant data have been available for *K. pneumonia*, although similar metabolites could be produced by *Klebsiella* could also affect the metabolism of *S. aureus.* On the other hand, the production of acetic acid and acetoin by *S. aureus* has been proposed as a potential mechanism affecting other bacteria in mixed cultures [40], which could be potentially responsible for the observed increased susceptibility of *K. pneumoniae* in dual-species biofilm. Nevertheless, the molecular mechanisms of susceptibility changes in *S. aureus*–*K. pneumoniae* mixed cultures remained largely unexplored. 

In addition to the impact of various metabolites on cellular physiology, in some studies changes in the permeability of biofilms formed by several bacteria have been reported [41,42]. Thus, low diffusion of both ampicillin and ciprofloxacin through *E. coli* and *K. pneumoniae* biofilms has been reported [43], and drastic reduction of bacterial susceptibility to ampicillin and vancomycin was observed also against *P. aeruginosa*–*S. aureus* dual-species biofilms [18]. By contrast, *S. aureus* biofilms were permeable for amikacin, ciprofloxacin, and cefotaxime, while also impenetrable for oxacillin and vancomycin [44]. In this work, the biofilm-diffusion tests revealed that the dual-species biofilm has increased permeability for ciprofloxacin, although remained impermeable for other antimicrobials under the conditions tested (Figure 4). As discovered by comparative analysis of extracellular matrix in mono- and dual-species communities, the ratio of the components alters considerably (Figure 5). In particular, there is a significant increase in the number of polysaccharides in the dual-species community of *S. aureus*–*K. pneumoniae* compared to their monomicrobial biofilms (Figure 5D), which apparently leads to the looser and more porous structure of the biofilm, as depicted by both SEM and CLSM (Figure 6). Fluorescent microscopy data also indicated that the distribution of polysaccharides in *S. aureus–K. pneumoniae* biofilms is rather heterogeneous, which is typical for the polysaccharide component of the extracellular matrix, which serves as a scaffold for bacterial biofilms and is capable of forming porous structures, as well as determines the efficacy of the diffuse barrier in protection from antimicrobials [45,46,47]. 

When the biofilm formation starts, bacteria change the gene expression pattern compared to free-living (planktonic) cells [48,49,50]. Thus, on the 8th h of growth *S. aureus* activates the expression of the *ica* operon required for the synthesis of polysaccharide intercellular adhesin (PIA) and poly-N-acetyl-1,6-β-glucosamine (PNAG), the dominant components of the *S. aureus* biofilm matrix [36,51]. In mixed communities, the level of *icaA* expression decreased compared to *S. aureus* monoculture (Figure 8A). By contrast, the expression of *pgaA* increased in *K. pneumoniae* in the dual-species community (Figure 8B). Thus, the relative expression of the *pgaA* gene was 10,000 times higher compared to the *icaA* gene, indicating that the synthesis of polysaccharides is provided by *K. pneumoniae* cells in mixed biofilms. Apparently, this may lead to loose biofilm structure formation, as it has been shown for *P. aeruginosa* when the biofilm is formed under the prevalent Pel polysaccharide secretion [52].

Apparently, the above changes in the number of polysaccharides in mixed communities could explain the higher permeability of the *S. aureus–K. pneumoniae* dual-species biofilms for ciprofloxacin in comparison with monomicrobial ones (Figure 4). Increased amounts of polysaccharides can contribute to the formation of a porous matrix, making the antibiotic capable of penetrating through the pores directly to the bacteria. The lack of growth inhibition with amikacin in this assay, apparently, could be attributed to the reduced diffusion rate of this antimicrobial in the studied model.

Remarkably, increased susceptibility to amikacin and ciprofloxacin was pronounced only in the biofilm and in detached cell clumps that occurred only for *K. pneumoniae*. Moreover, the susceptibility of *S. aureus* to ampicillin, vancomycin, and ceftazidime in cell clumps detached from dual-species biofilms was similar to that in the biofilm (See Figure 3), assuming that cells are protected by the residual extracellular matrix. These data are in agreement with decreased permeability of *K. pneumoniae* and *S. aureus–K. pneumoniae* biofilms at least for ceftazidime (Figure 4E) and no permeability for amikacin, vancomycin, and ampicillin (Figure 4). A similar effect of increased resistance has been reported for mixed communities of *P. aeruginosa*–*S. aureus* [18] and *P. aeruginosa*–*P. protegens*–*K. pneumoniae* [32]. Furthermore, in multi-species biofilm consisting of *P. aeruginosa*, *S. aureus*, *K. pneumonia,* and *A. baumannii* resistance to ciprofloxacin has been increased mainly due to the tolerance of *K. pneumoniae* [28], confirming that the biofilm matrix significantly affects the efficiency of antimicrobial treatment, while depending on bacterial community counterparts. The molecular mechanisms of these effects remain unexplored, and the directions of susceptibility alterations are variable and apparently should be tested for individual communities to optimize the treatment scenarios.

## 4. Materials and Methods

### 4.1. Bacterial Strains and Growth Media

*Staphylococcus aureus* subsp*. aureus* (ATCC^®^ 29213™) and *Klebsiella pneumoniae* (clinical isolate obtained from the Kazan Institute of Epidemiology and Microbiology, Kazan, Russia) were used in the study. Bacteria were stored as a 50% glycerol stock at −80 °C and maintained on the LB medium. Biofilms were grown for 48 h in the BM broth (glucose 5g, peptone 7 g, MgSO_4_ × 7H_2_O 2 g, and CaCl_2_ × 2H_2_O 0.05 g in 1.0 L tap water) [53,54,55]. The mannitol salt agar (peptone 10 g, meat extract 1 g, NaCl 75 g, D-mannitol 10 g, agar-agar 12 g in 1.0 L tap water) and Endo agar (BD Diagnostics) were used for the differential count of *S. aureus* and *K. pneumoniae*, respectively.

### 4.2. Determination of Minimum Inhibitory Concentration and Minimum Bactericidal Concentration

The minimum inhibitory concentration (MIC) was determined by microdilution method in BM broth according to EUCAST recommendations [56]. Antibiotics were diluted with broth in 96-well plastic plates (Eppendorf Cell Culture Plates) at concentrations of 0.25–512 μg/mL. The wells were inoculated with 200 μL of bacterial culture (2–9 × 10^6^ CFUs/mL) and incubated at 37 °C under static conditions. The minimum inhibitory concentration was defined as the lowest concentration of a substance providing no bacterial growth during 24 h of incubation.

The minimum bactericidal concentration (MBC) was defined as the lowest concentration of a substance providing the reduction of viable cells by factor 1000 during 24 h of incubation. For this, 1000× culture dilutions were made from MIC-testing wells with no visible growth, inoculated into fresh broth, and incubated for 24 h. The MBC was taken as the lowest concentration of the substance providing no bacterial growth during 24 h after inoculation.

### 4.3. Biofilm Assays

The total biomass of biofilms was assessed in 24-well polystirol plates by staining with crystal violet as described earlier in [57] with modifications. Bacteria were seeded in 1 mL of BM broth at initial density of 1–5 × 10^7^ CFU/mL and grown at 37 °C for 48 h under static conditions. Then wells were washed once with sterile PBS, air-dried for 20 min, and stained with 1 mL of crystal violet (0.5% solution in 96% ethanol) for 20 min. Wells were washed three times with PBS and dried. The bound dye was eluted in 1 mL of 96% ethanol, and the absorbance was read on a Tecan Infinite 200 Pro microplate reader (Switzerland, Männedorf) at 570 nm.

Alternatively, the amount of exopolymeric matrix of biofilms was evaluated by Congo red depletion assay [58] with modifications. For that, 48 h old biofilms were washed and suspended in fresh LB broth containing Congo red at final concentration of 80 μg/mL. After 90 min incubation at 37 °C, the biomass was removed by centrifugation for 5 min at 4400 rpm, the supernatant was transferred to 96-well plates and amount of residual Congo red was measured on microplate reader at 490 nm. The Congo red depletion was calculated as difference in the absorption of initial solution and solutions incubated with biofilms.

### 4.4. CFUs Counting

To assess the viability of bacteria after exposure to antimicrobials, they were grown in 24-well plastic adhesive plates for 48 h at 37 °C without shaking. After that, the medium was replaced with a fresh one, the test substances were added and incubated for 24 h. Next, serial 10-fold dilutions of the culture liquid were prepared in 0.9% NaCl, and five μL of the suspension was transferred to plates with either mannitol salt agar or Endo agar to differentiate *S. aureus* and *K. pneumoniae*, respectively, in mixed cultures. Plates were incubated for 24 h at 37 °C and CFUs were counted from the last drops containing 5–15 colonies. Further, biofilms were washed with sterile 0.9% NaCl, mechanically suspended in 0.9% NaCl with subsequent treatment in an ultrasonic bath for 2 min to facilitate the disintegration of bacterial clumps [59] and CFUs were counted as described above. Data were presented as median and IQR from three independent experiments with three technical repeats in each.

### 4.5. The Quantification of Matrix Composition

The content of eDNA, proteins, and polysaccharides in the biofilm matrix was assessed by differential fluorescent staining with SYBRGreen (1:5000 dilution of commercial stock, eDNA stain), Sypro Orange (ready to use ×1000 solution, proteins stain), ConA-TMR (500 mg/mL, α-polysaccharides stain), Calcofluor White M2R (CFW) (1 mg/mL, β- polysaccharides stain). All dyes were purchased from Sigma. The 48 h old biofilms were established in 96-well black plates and washed once with PBS solution to remove non-adherent cells. Then dyes were added (100 µL per well) and incubation was followed for 15 min at 37 °C. Then, wells were washed with PBS, filled with 100 µL of PBS, and the fluorescence was measured on a microplate reader Tecan Infinite 200 Pro (Switzerland) at the desired wavelengths (see Table 2).

To assess the distribution of components in the matrix of biofilm, microscopy was performed using an Olympus IX83 inverted microscope supplemented with a STEDYCON ultrawide extension platform. Both mono- and mixed cultures of *S. aureus* and *K. pneumoniae* were grown on cell imaging cover slips (Eppendorf) under static conditions for 48 h in BM broth. After that, the culture liquid was discarded, and fluorescent dyes were added at the same concentrations as described earlier and incubated for 15 min at 37 °C. 

### 4.6. Microscopy

The ultrastructure of mono- and dual-species biofilms was assessed with scanning electron microscopy. Biofilms were established by seeding the bacteria in BM broth in 34 mm plastic adhesive Petri dishes (TC-treated, Eppendorf, 2 mL per plate) followed by 48 h growth at 37 °C under static conditions. Mature biofilms were washed 3 times with water and fixed with glutaraldehyde (1% water solution) for 24 h. After subsequent washing with deionized water, the plates were dried for 12 h at 55 °C and coated in vacuum with gold on SCD 004 (Balzers AG, Balzers, Liechtenstein). SEM was performed on Quanta 200 microscope (FEL Company, Skokie, IL, USA) at 29 kV in the Ultramicroanalysis Research Center at the Limnological Institute of the Siberian Branch of the Russian Academy of Sciences, Irkutsk. Additionally, for analysis of structure of mono- and dual-species biofilms confocal laser scanning microscopy was used. Mature 48 h biofilms on cell imaging coverslips (Eppendorf) were stained by DAPI (5 mg/mL) and analyzed by Olympus IX83 inverted microscope supplemented with a STEDYCON ultrawide extension platform. 

To analyze the distribution of extracellular matrix components, mono- and two-species biofilms were stained with fluorescent dyes SYBRGreen, Sypro Orange, ConA-TMR, Calcofluor White M2R (CFW) as described previously and CLSM was performed using an Olympus IX83 inverted microscope supplemented with a STEDYCON ultrawide extension platform.

### 4.7. Penetration of Antimicrobials Throw Biofilm Matrix

The assay was performed as described in [44] with slight modifications. To obtain mono- and dual-species biofilms, bacterial suspension with an optical density of 3 × 10^7^ CFU/mL in BM broth was dropped on the surface of sterile nitrocellulose discs. Then, the discs were placed onto LB-agar and incubated for 48 h at 37 °C to let the biofilm grow on the disc. After, discs were transferred onto fresh LB-agar containing desired antibiotic at a concentration corresponding to 1×MBC against given bacterium (See Table 2). On the top of the biofilm, a new nitrocellulose disc and then a Whatmann disc were placed and incubation was continued for 24 h at 37 °C to allow the antibiotic to diffuse through the biofilm and accumulate in the paper disc. Then, the paper discs were placed onto plates seeded with bacterial lawn and incubated for 24 h at 37 °C. As a negative control, the agar plates without antibiotics were used in the second step. For the positive control, all manipulations were made with sterile membranes without biofilm. The permeability of the biofilm for antibiotics was evaluated by measuring the bacterial growth repression zone.

### 4.8. RNA Isolation and Real-Time One-Step qRT-PCR

To assess the expression of genes responsible for synthesis of the polysaccharide component of the extracellular matrix, the quantitative RT-PCR was performed on BioRad CFX96 amplifier (BioRad, Singapore) using Extra Mix for reverse transcription and quantitative real-time PCR in a one-step method (BioLabMix, Novosibirsk, Russia) with SYBR Blue under conditions recommended by the manufacturer and product detection at FAM filterset. The oligonucleotides used for the qRT-PCR are shown in Table 3. A total RNA was extracted from 12 h biofilms. Reaction mixture (50 µL) contained 1× qRT-PCR SYBR Blue buffer, 0.1 µM of each primer, 0.1 μM of each dNTP, 2.5% DMSO, 5% qRT-PCR extra-mix and nuclease-free water (DEPC). The qRT-PCR program included reverse transcription at 45 °C for 30 min, followed by 37 cycles of primer annealing–elongation–melting. Primer annealing temperatures were calculated using the Tm Calculator service (https://tmcalculator.neb.com, accessed on 1 November 2022). The 16s rRNA and *proC* genes were used as references for *S. aureus* and *K. pneumoniae*, respectively; the transcription level of *icaA* gene was normalized by the 16 s rRNA transcription level and *pgaA* gene was normalized by the *proC* rRNA transcription level.

### 4.9. Statistical Analysis

Experiments were carried out in three biological repeats with newly prepared cultures and medium in each of them. The statistical significance of the discrepancy between monoculture and mixed biofilms treatment efficacy was determined using the non-parametric one-way analysis of variance (Kruskal–Wallis) test with a significance level of *p* < 0.05.

## 5. Conclusions

Taken together, our data indicate significant alterations in the antimicrobial efficiency in mixed biofilms of *S. aureus*–*K. pneumoniae* in comparison with mono-species biofilms of each counterpart. These alterations are associated with changes in the biofilm-associated gene expression pattern, in turn leading to alterations in both biofilm structure and properties, as well as in bacterial susceptibility to antimicrobials. On the other hand, even in cell clumps detached from the dual-species biofilm, residual biofilm provides protection, thus making *S. aureus* insensitive to vancomycin, ampicillin, and ceftazidime. In turn, increased efficiency of amikacin and ciprofloxacin against both bacteria could be observed in comparison with mono-species biofilms of each counterpart. While the molecular triggers of these changes remain undiscovered, detailed knowledge about the changes in the antibiotic susceptibility to given drugs opens doors for corrections of the treatment of infections associated with *S. aureus*–*K. pneumoniae* biofilm-associated infections.

## Figures and Tables

**Table 1:** Minimum inhibitory (MIC) and bactericidal (MBC) concentrations of antibiotics, µg/mL.

	*S. aureus*		*K. pneumoniae*	
	MIC	MBC	MIC	MBC
Amikacin	8	32	4	4
Ceftazidime	8	32	32	256
Ciprofloxacin	0.25	16	0.5	0.5
Ampicillin	0.25	2	ND	ND
Vancomycin	2	64	ND	ND

**Table 2:** The emission and excitation wavelengths of fluorescent dyes for differential staining of biofilms components.

Dye	SYBR Green	Sypro Orange	ConA-TMR	CFW
Excitation Wavelength	497 nm	470 nm	552 nm	254 nm
Emission Wavelength	520 nm	570 nm	578 nm	432 nm
Target	eDNA	Proteins	α-polysaccharides	β-polysaccharides

**Table 3:** The oligonucleotides used for the qRT-PCR.

Primer	Nucleotide Sequence	Reference
Kp-pgaA-for	5′ CACCTGCAGACGCTCTCCTATGTC 3′	This work
Kp-pgaA-rev	5′ AAGAGGAGATGACCCAGCCGATG 3′	This work
proC-for	5′ GATTGCCGATATCGTCTTCG 3′	[60]
proC-rev	5′ GAGACCACCAGCGACTCTTT 3′	[60]
icaA-for	5′ AAGCCAACGCACTCAATCAAGG 3′	[61]
icaA-rev	5′ GGATTACCTGTAACCGCACCAAG 3′	[61]
16s rRNA-for	5′ GGGACCCGCACAAGCGGTGG 3′	[61]
16s rRNA-rev	5′ GGGTTGCGCTCGTTGCGGGA 3′	[61]

## Data Availability

All data are available in the manuscript file.
